# Revision Quadriceps Tendon Rupture Repair Using BioBrace Augment: A Novel Case and Technique

**DOI:** 10.7759/cureus.97868

**Published:** 2025-11-26

**Authors:** Samora Maranya, Mitchell Baker, Nathan Urquhart

**Affiliations:** 1 Orthopaedics, Dalhousie University, Halifax, CAN; 2 Hospital Medicine, Dalhousie University, Halifax, CAN; 3 Orthopaedic Surgery, Dalhousie University, Halifax, CAN

**Keywords:** augment, biobrace, quadriceps, reconstruction, revision, tendon rupture

## Abstract

Quadriceps tendon retears are a challenging injury to repair/reconstruct, resulting from eccentric traumatic knee events. Chronic tears or failed reconstructions have stretched out or attenuated tissue that needs to be excised to regain lag-free knee extension after repair. To create a strong repair, augmentation with either an allograft or an autograft is often required. We propose the use of bio-composite material BioBrace® (ConMed, New Haven, Connecticut) to augment the repair, giving both structural support immediately after repair and biological induction of host tissue into the graft as it gradually resorbs. We describe a repair and augment technique that is easily reproducible.

## Introduction

Quadriceps tendon rupture is an uncommon but severe injury, with a reported annual incidence of 1.37 per 100,000 people [1]. It occurs as a result of sudden eccentric loading of the quadriceps tendon, most frequently in older adults and those with comorbidities such as rheumatoid arthritis, gout, diabetes, and previous steroid use [2]. Males tend to be affected at a higher rate than females, with a ratio of 8:1 [3].

Ruptures of the quadriceps tendon are typically repaired surgically (complete tears) with either transosseous sutures or suture anchors. Suture anchor use was first described by Maniscalco et al., with reported benefits including shorter operative times, smaller incisions, and less disruption of the local vasculature [4]. A meta-analysis comparing the two techniques found that transosseous sutures produce marginally better outcomes in terms of less extensor lag [5,6]. The literature shows a postoperative complication rate of 18%, with re-rupture requiring revision surgery occurring in 11% of cases. No statistical differences in outcomes have been observed between the two techniques [7].

We present a case report and technique description using a standard transosseous revision repair augmented by a novel biologic implant, BioBrace® (ConMed, New Haven, Connecticut), composed of ultra-porous type I collagen, allowing induction and ingrowth of new tissue, and bio-resorbable poly(L-lactide) (PLLA) microfilaments for increased mechanical strength. Its use has been described in the management of rotator cuff tear repair, anterior cruciate ligament reconstruction augmentation, and Achilles tendon repair [8,9]. The advantage of BioBrace® is that it provides structural strength at time zero and stimulates ingrowth of native tissue during its resorption [10,11]. It therefore combines properties of both biology and strength into a single implant.

## Case presentation

**Figure 1:**
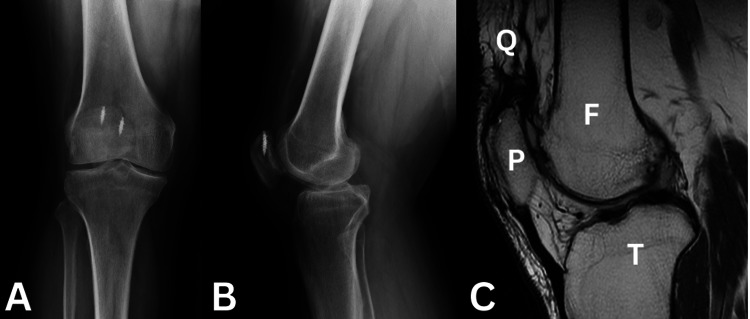
(A) Anteroposterior and (B) lateral X-ray radiographs showing suture anchors in the superior patella pole. (C) A T1 sagittal MRI of the knee with Q showing the quadriceps tear, P showing the patella, F showing the femur, and T showing the tibia.

We present a case of a 55-year-old patient who sustained a fall with re-rupture of his quadriceps tendon that went undiagnosed for eight months, during which time he used a cane to assist with walking. His previous injury involved a ground-level fall on slippery ground, causing sudden eccentric loading at the knee. He underwent primary repair of his injury using metallic suture anchors to his proximal patella pole with no augmentation. For this injury, the patient had a palpable gap proximal to the patella, moderate quadriceps atrophy, and an extensor lag of 35°. Anteroposterior and lateral X-ray radiographs of the knee showed anchors in the superior patella pole from a previous quadriceps tendon repair (Figures 1A, 1B) but no fracture or soft tissue anomaly. However, the sagittal MRI views demonstrated a high-grade partial quadriceps tear (Figure 1C).

Preoperative planning

A history and physical examination of the knee was performed to confirm the presence of a quadriceps retear, revealing an extension lag and, occasionally, a palpable gap. Imaging, including plain X-ray radiographs and magnetic resonance imaging, was procured, examining the presence/absence of fractures as well as detailed characterization of the tear (location, size, and retraction). Revision repair was indicated in this case of identified retear that correlated with physical exam findings.

Surgical technique

Step 1: Patient Positioning

The patient was positioned supine with a foam wedge pillow. A tourniquet was applied but not inflated; the lower limb was prepped with chlorhexidine and draped in a sterile fashion. A single-use sterile marker was used to mark out the proximal patella pole and longitudinal incision proximal to it.

Step 2: Approach to Revision Quadriceps Tendon

**Figure 2:**
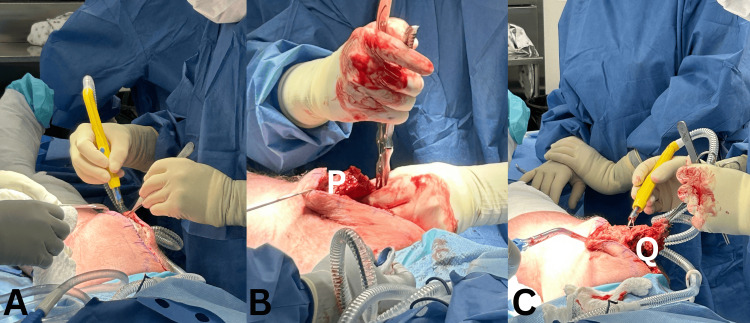
(A) Midline approach proximal to and including the superior patella pole, raising thick skin flaps. (B) Quadriceps tendon and superior patella pole (P) preparation. (C) Freshening edges and removal of callus/scar tissue from quadriceps (Q).

A midline incision was made at the previous scar, raising thick flaps on both sides (Figure 2A). The stretched-out area of the rupture was identified, and the medial and lateral retinacula were visualized.

Step 3: Tendon Preparation

With the lower limb fully extended, an assessment was made as to whether the native tendon could be approximated to the proximal patella pole without excessive tension while eliminating any extension lag. Any redundant/stretched-out tendon or scar tissue was excised by making a V-shaped excision of the excess tissue on the medial and lateral aspects of the knee just proximal to the patella. This represented the area that is typically torn during the previous quadriceps tendon rupture.

A rongeur was used to freshen up the edge of the superior patella pole (Figure 2B), and scar tissue was removed sharply from the quadriceps (Figure 2C). The newly created medial and lateral retinacula incisions were then whipped with No. 5 Ethibond suture (Ethicon, Somerville, New Jersey).

Step 4: Tunnel Drilling Through Patella and Suture Passage

**Figure 3:**
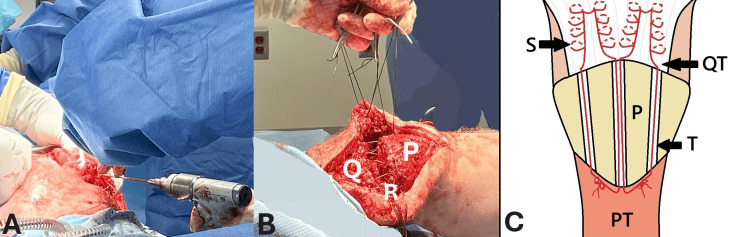
(A) Drilling of three intraosseous tunnels from proximal to distal patella pole. (B) Krakow stitch through the quadriceps tendon (Q) and repair of retinacula (R), with the patella (P) in view. (C) Diagram showing the repair through intraosseous tunnels (S = suture; QT = quadriceps tendon; P = patella; T = tunnel; PT = patella tendon).

A 2.5 mm drill was then used to drill three parallel tunnels from the superior to the inferior patella pole (Figure 3A). Two high-strength No. 5 Hi-Fi® (ConMed, Largo, Florida) sutures were used to suture the quadriceps tendon using the Krackow technique, and the needle was then removed. The central tunnel had the two central suture ends passed through, and the medial and lateral holes had a single strand each. The retinacula sutures were then tied sequentially, followed by the quadriceps repair sutures at the inferior patella pole with the knee in full extension and tendon in contact with the superior patella pole (Figure 3B). A schematic representation of the construct is shown in Figure 3C.

Step 5: BioBrace® Augment Graft Fixation and Closure

**Figure 4:**
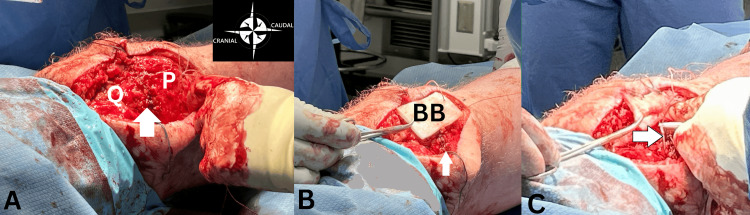
(A) Complete approximation of the quadriceps tendon and retinacula repair (white arrow) (Q = quadriceps tendon; P = patella). (B) BioBrace® (BB) (40 x 60 mm) soaked in saline and placed longitudinally. (C) Graft fixation centered at the tendon/superior pole insertion, sutured onto the tendon (white arrow).

After a complete approximation of the quadriceps tendon and retinacula repair (Figure 4A), the BioBrace® bio-composite augment measuring 40 x 60 mm that had been soaked in saline was then placed longitudinally across the repaired defect (Figure 4B). This orientation was important as it placed the tissue fibers in the direction that provides the most strength. The graft fixation was centered at the tendon/superior pole insertion (Figure 4C), sutured onto the tendon.

**Figure 5:**
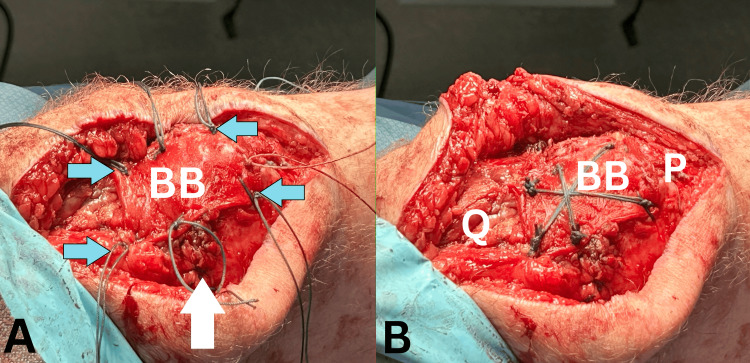
(A) Six-point fixation (blue arrows) of the graft (BioBrace®, BB) with No. 5 ETHIBOND EXCEL™ suture. (B) Stellate repair with ties across the graft, pushing it onto the quadriceps tendon (Q) and patella (P).

Fixation at six points on the graft was performed using No. 5 ETHIBOND EXCEL™ (Ethicon) with sutures passed through the quadriceps tendon with a mattress technique (Figure 5A). The suture ends were then tied across the graft, further securing it onto the tendon (Figure 5B). The steps in this repair have been outlined in Table 1.

**Table 1:** Steps in revision quadriceps tendon rupture repair using BioBrace augmentation.

Steps	Technique
Step 1	Patient positioned supine with a foam wedge pillow. A tourniquet is applied but not inflated. Marking out the proximal patella pole and longitudinal incision proximal to it.
Step 2	A midline incision and raising thick flaps medially and laterally. The rupture area, medial and lateral retinacula, is identified.
Step 3	Excision of excessive scarred quadriceps tendon tissue by making a V-shaped incision. Whipping of medial and lateral retinacula incisions.
Step 4	Drilling of three longitudinal transosseous tunnels from proximal to distal patella pole (2.5 mm drill). Two high-strength No. 5 suture whipped into the quadriceps tendon (Krakow method). Passage of whipped suture into transosseous tunnels (2 limbs in the central tunnel, 1 limb in the medial and lateral tunnels). Repair of retinacula incisions completed. Tying of quadriceps sutures at the inferior patella pole.
Step 5	BioBrace graft (40 x 60 mm) longitudinally placed and centered over the repair. Six-point fixation with No. 5 Ethibond suture (Mattress technique). Suture limbs tied across the graft to the longitudinally opposite limbs.

## Discussion

Patients with quadriceps tendon re-rupture often present with significant limitations in active knee extension. The re-ruptured tendon is frequently shortened and of poor quality, especially in chronic tears, resulting in poorer outcomes compared to primary repair. A systematic review by Oliva et al showed a re-rupture rate of 10% with an extension lag of 5° observed in 22% of revision chronic tear cases [12-14].

This injury predominantly affects men aged between 50 and 60 years and involves eccentric loading forces during a fall [15,16]. Tears can easily be missed, leading to delayed diagnosis with chronic tears producing fibrotic, retracted scars that require grafting to bridge the defect [17].

There have been numerous repair techniques described in the literature involving transosseous vs. suture anchor methods. Additionally, a myriad of reconstructive methods using autograft/allograft exist. The use of BioBrace serves to induce host tissue ingrowth onto the end-to-end repair, providing both biological and structural properties from time point zero.

Despite advances in surgical techniques, complications such as re-rupture remain a significant concern in patients with comorbidities such as diabetes, chronic kidney disease, rheumatoid arthritis, and obesity. Bio-inductive augmentation is an emerging strategy in tendon repair that shows promise in aiding tendon healing in massive rotator cuff tears [18]. Conventional bio-inductive materials encourage new tissue formation but lack mechanical integrity, while human allografts offer structural strength without the same degree of inductive potential [10]. The advantages and disadvantages of using this technique have been outlined in Table 2.

**Table 2:** Advantages and disadvantages of the technique.

Advantages
BioBrace® (40 x 60 mm) is used as packaged with no adjustments
The technique is easily reproducible
Repair is tension-free with the knee in full extension during augmentation
Augmentation provides initial strength and potential for host tissue ingrowth
Disadvantages
Potential (low) risk of induction of host inflammatory response to graft and subsequent infection
High cost
Not ideal if the quadriceps tendon requires reconstruction with an allograft of V-Y advancement to bridge a defect rather than reinforce a repair as described

## Conclusions

The patient was followed up in the clinic, having been partially weight-bearing on crutches, with no wound-healing problems. He demonstrated the ability to perform a straight-leg raise with no extension lag at the six-week mark postoperatively. There was no palpable gapping in his extensor mechanism.

We hope this case provides insight into the challenges of repair of chronic quadriceps tendon tear re-rupture and the importance of adherence to sound surgical principles, as well as the use of available biological augments to improve strength and tendon healing. A robust fixation enables early mobilization and weight-bearing, potentially reducing the occurrence of knee stiffness and enabling the early start of a quadriceps strengthening program.
